# Factors Associated with Failure of Reperfusion in Endovascular Therapy for Acute Ischemic Stroke: A Multicenter Analysis

**DOI:** 10.1007/s00062-020-00880-8

**Published:** 2020-02-17

**Authors:** Fabian Flottmann, Gabriel Broocks, Tobias Djamsched Faizy, Rosalie McDonough, Lucas Watermann, Milani Deb-Chatterji, Götz Thomalla, Moriz Herzberg, Christian H. Nolte, Jens Fiehler, Hannes Leischner, Caspar Brekenfeld, for the GSR investigators, Tobias Boeckh-Behrens, Silke Wunderlich, Arno Reich, Martin Wiesmann, Ulrike Ernemann, Till-Karsten Hauser, Eberhard Siebert, Sarah Zweynert, Georg Bohner, Alexander Ludolph, Karl-Heinz Henn, Waltraud Pfeilschifter, Marlis Wagner, Joachim Röther, Bernd Eckert, Jörg Berrouschot, Albrecht Bormann, Christian Gerloff, Elke Hattingen, Gabor Petzold, Sven Thonke, Christopher Bangard, Christoffer Kraemer, Martin Dichgans, Frank Wollenweber, Lars Kellert, Franziska Dorn, Moriz Herzberg, Marios Psychogios, Jan Liman, Martina Petersen, Florian Stögbauer, Peter Kraft, Mirko Pham, Michael Braun, Gerhard F. Hamann, Andreas Kastrup, Christian Roth, Klaus Gröschel, Timo Uphaus, Volker Limmroth

**Affiliations:** 1 Department of Diagnostic and Interventional Neuroradiology, University Medical Center Hamburg-Eppendorf, Haus Ost 22 (O 22), Martinistr. 52, 20246 Hamburg, Germany; 2 Department of Neurology, University Medical Center Hamburg-Eppendorf, Hamburg, Germany; 3 Department of Neuroradiology, University Hospital, LMU Munich, Munich, Germany; 4 Department of Neurology, Charité—Universitätsmedizin Berlin, Berlin, Germany; 5 Center for Stroke Research Berlin, Charité—Universitätsmedizin Berlin, Berlin, Germany

**Keywords:** Endovascular treatment, Large-vessel occlusion, Mechanical thrombectomy, Unsuccessful reperfusion, Registry study

## Abstract

**Aim:**

In acute large vessel occlusions, endovascular therapy (EVT) achieves flow restoration in the majority of cases; however, EVT fails to achieve sufficient reperfusion in a substantial minority of patients. This study aimed to identify predictors of failed reperfusion.

**Methods:**

In this study 2211 patients from the German Stroke Registry who received EVT for anterior circulation stroke were retrospectively analyzed. Failure of reperfusion was defined as thrombolysis in cerebral infarction (TICI) grades 0/1/2a, and sufficient reperfusion as TICI 2b/3. In 1629 patients with complete datasets, associations between failure of reperfusion and baseline clinical data, comorbidities, location of occlusion, and procedural data were assessed with multiple logistic regression.

**Results:**

Failure of reperfusion occurred in 371 patients (16.8%) and was associated with the following locations of occlusion: cervical internal carotid artery (ICA, adjusted odds ratio, OR 2.01, 95% confidence interval, CI 1.08–3.69), intracranial ICA without carotid T occlusion (adjusted OR 1.79, 95% CI 1.05–2.98), and M2 segment (adjusted OR 1.86, 95% CI 1.21–2.84). Failed reperfusion was also associated with cervical ICA stenosis (>70% stenosis, adjusted OR 2.90, 95% CI 1.69–4.97), stroke of other determined etiology by TOAST (Trial of ORG 10172 in acute stroke treatment) criteria (e.g. nonatherosclerotic vasculopathies, adjusted OR 2.73, 95% CI 1.36–5.39), and treatment given outside the usual working hours (adjusted OR 1.41, 95% CI 1.07–1.86). Successful reperfusion was associated with higher Alberta stroke program early CT score (ASPECTS) on initial imaging (adjusted OR 0.85, 95% CI 0.79–0.92), treatment with the patient under general anesthesia (adjusted OR 0.72, 95% CI 0.54–0.96), and concomitant ICA stenting in patients with ICA stenosis (adjusted OR 0.20, 95% CI 0.11–0.38).

**Conclusion:**

Several factors are associated with failure of reperfusion, most notably occlusions of the proximal ICA and low ASPECTS on admission. Conversely, stent placement in the proximal ICA was associated with reperfusion success.

**Electronic supplementary material:**

The online version of this article (10.1007/s00062-020-00880-8) contains supplementary material, which is available to authorized users.

## Introduction

Endovascular therapy (EVT) has become established as the standard of care in acute stroke due to large vessel occlusions and leads to sufficient reperfusion in the majority of cases [1]; however, EVT fails to achieve sufficient reperfusion in a substantial minority of anywhere between 12–41% of patients [1, 2].

Reasons for reperfusion failure have been investigated in single center studies on a procedural level (e.g. analyzing anatomical and technical difficulties) and have been classified into technical obstacles in reaching the occlusion site vs. failure to remove the clot [3, 4]. Furthermore, EVT failure has been found to be associated with advanced patient age and distal occlusions [4].

It is unknown which other factors could be associated with reperfusion failure in EVT. It was hypothesized that predictors of failed reperfusion could be identified using a large multicenter cohort of EVT patients, which includes baseline clinical data, admission imaging, location of occlusion, time of treatment, and thrombectomy technique.

## Material and Methods

### Patient Selection

Available data of patients enrolled in the German Stroke Registry—Endovascular Treatment (GSR-ET 07/2015-04/2018; ClinicalTrials.gov Identifier: NCT03356392) between June 2015 and April 2018 were included. The GSR-ET is an ongoing, open-label, prospective, multicenter registry of 25 primary stroke centers in Germany, prospectively collecting consecutive patients undergoing EVT [5, 6].

The inclusion criteria for the present study were: 1) acute ischemic stroke due to large vessel occlusion in the anterior circulation in patients >18 years old, 2) decision to perform EVT and 3) available data on final thrombolysis in cerebral infarction (TICI) grade following angiography. Study protocols and procedures were conducted in compliance with the Declaration of Helsinki and in accordance to ethical guidelines (the leading ethics committee of the Ludwig-Maximilians University Munich approved the GSR-ET and the study obtained additional approval from the local ethics committees of the participating hospitals).

### Data Acquisition

Data acquisition was performed according to the GSR-ET protocol: data were collected by the treating physicians and entered into a standardized form using a secure web environment. Baseline demographics, comorbidities, admission National Institutes of Health Stroke Scale (NIHSS) score, modified Rankin scale before admission (pre-mRS), and administration of intravenous thrombolysis were documented as well as periprocedural complications, time intervals between symptom onset (if available), admission, groin puncture, and flow restoration.

### Image Analysis

Baseline Alberta stroke program early CT score (ASPECTS) was determined on preintervention non-enhanced computed tomography (CT) scans or diffusion-weighted imaging (in the case of magnetic resonance imaging, MRI). The location of the occlusion was assessed on an initial angiographic series, and ICA occlusions were categorized into occlusions of the cervical ICA, the intracranial ICA without carotid T involvement and of the carotid T. Cervical ICA stenosis was defined as stenosis of more than 70% of the cervical segment of the ICA and evaluated according to clinical routine, which follows the German guideline recommendations to apply the NASCET criteria [7].

Reperfusion failure was defined as no, minimal or partial reperfusion (defined by TICI 0, 1 and 2a, respectively), as opposed to sufficient reperfusion (TICI 2b-3).

### Statistical Analysis

All analyses were performed with R software version 3.5.1 (R Foundation, Vienna, Austria) [8]. Normally distributed variables are displayed as mean and standard deviation. Non-normally distributed variables are displayed as median and interquartile range. Categorical variables are reported as proportions. A multiple logistic regression for failure of reperfusion was performed, including the following independent variables after a priori selection: age, sex, pre-admission mRS, admission NIHSS, admission ASPECTS, comorbidities (arterial hypertension, diabetes mellitus, dyslipidemia, smoking), i.v. thrombolysis, location of occlusion (side and occluded vessel), type of anesthesia, ICA stenosis, concomitant ICA stenting, type of EVT, etiology, and whether treatment was performed during regular clinical routine. A *p* value <0.05 was considered statistically significant.

## Results

### Baseline Characteristics

**Fig. 1:**
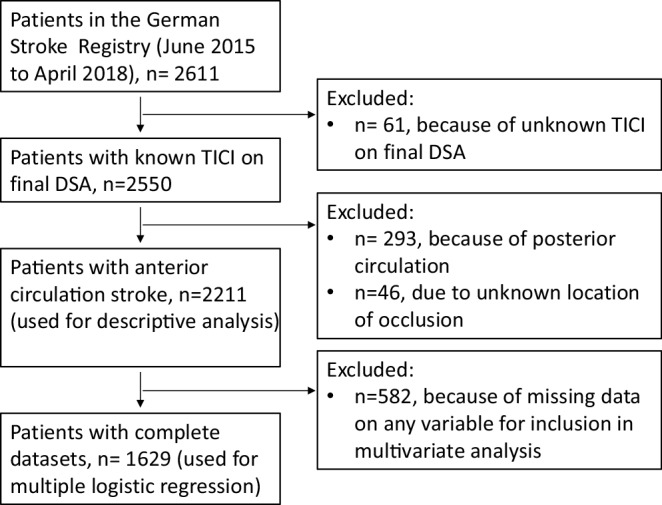
Patient selection flow chart. *TICI* Thrombolysis in cerebral infarction, *DSA* digital subtraction angiography

A total of 2611 patients were identified in the complete dataset, 2211 of which fulfilled the inclusion criteria (Fig. 1). Among these 51.6% were female and the mean age was 72.6 (±13.0) years (Table 1). The median NIHSS score on admission was 15 (range 10–19). The median ASPECTS on admission imaging was 9 (range 7–10). Concomitant stenting of cervical ICA stenosis was performed in 243 cases (11.0%). Failure of reperfusion occurred in 371 patients (16.8%, Table 2).

**Table 1:** Baseline clinical data

Variable	Valid data (*n*, %)	Sufficient reperfusion (TICI 2b‑3, *n* = 1840)	Insufficient reperfusion (TICI 0/1/2a, *n* = 371)
*Age, years (mean, SD)*	2211 (100%)	72.6 (12.9)	72.9 (13.5)
*Female sex, n (%)*	2210 (100%)	945 (51.4%)	195 (52.6%)
*Hypertension, n (%)*	2192 (99.1%)	1383 (75.9%)	271 (73.4%)
*Diabetes mellitus, n (%)*	2195 (99.3%)	394 (21.6%)	72 (19.6%)
*Dyslipidemia, n (%)*	2187 (98.9%)	639 (35.1%)	106 (28.8%)
*Atrial fibrillation, n (%)*	2188 (99%)	811 (44.5%)	124 (33.8%)
*Initial NIHSS score (median, Q1–Q3)*	2190 (99.1%)	15 (10–18)	15 (11–19)
*Initial ASPECTS (median, Q1–Q3)*	1906 (86.2%)	9 (7–10)	8 (7–9)
*Initial occlusion site, n (%)*	2211 (100%)	–	–
*Left hemisphere*	–	928 (50.5%)	205 (55.4%)
*Location of vessel occlusion*	2211 (100%)	–	–
ICA (cervical)	–	90 (4.89%)	32 (8.63%)
Intracranial ICA (without Carotid T)	–	106 (5.76%)	38 (10.2%)
Intracranial ICA (Carotid T)	–	346 (18.8%)	84 (22.6%)
Middle cerebral artery, M1 proximal	–	745 (40.5%)	131 (35.3%)
Middle cerebral artery, M1 distal	–	410 (22.3%)	77 (20.8%)
Middle cerebral artery, M2 segment	–	406 (22.1%)	97 (26.1%)
*Intravenous thrombolysis, n (%)*	–	152 (60.6%)	29 (52.7%)
*Onset to admission, min (median, Q1–Q3)*	1338 (60.5%)	128 (60.0–200)	140 (59.5–208)
*Treatment out of daytime routine (Monday to Friday, 8 a.m. to 5 p.m.)*	2137 (96.7%)	688 (38.7%)	156 (43.6%)
*Stroke etiology*	2180 (98.6%)	–	–
Cardioembolism	–	993 (54.7%)	160 (44.0%)
Dissection	–	34 (1.87%)	9 (2.5%)
Atherosclerosis	–	415 (22.9%)	112 (30.8%)
Other determined etiology	–	68 (3.74%)	26 (7.14%)
Unknown etiology	–	305 (16.8%)	57 (15.7%)

*ASPECTS* Alberta stroke program early CT score, *ICA* Cervical Internal Carotid Artery, *NIHSS* National Institutes of Health Stroke Scale, *SD* standard deviation

**Table 2:** Procedural and clinical outcome

Variable	Valid data (*n*, %)	Sufficient reperfusion (TICI 2b‑3, *n* = 1840)	No, minimal reperfusion or partly reperfusion (TICI 0/1/2a, *n* = 371)
*Final TICI score*	2211 (100%)	–	–
*0*	–	0 (0.00%)	193 (52.0%)
*1*	–	0 (0.00%)	39 (10.5%)
*2a*	–	0 (0.00%)	139 (37.5%)
*2b*	–	829 (45.1%)	0 (0.00%)
*3*	–	1011 (54.9%)	0 (0.00%)
*Cervical ICA stenosis >70% on angiogram, n (%)*	2093 (94.7%)	268 (15.4%)	75 (21.2%)
*Concomitant stenting of the cervical ICA*	2211 (100%)	210 (11.4%)	33 (8.89%)
*No thrombectomy maneuver performed (spontaneous reperfusion or failure to access thrombus)*	1943 (87.8%)	31 (1.9%)	57 (18.2%)
*Time from admission to groin puncture (median, Q1–Q3)*	2086 (94.3%)	69 (45–99)	76 (50–112)
*Time from groin puncture to final TICI (median, Q1–Q3)*	1724 (78%)	115 (82–154)	133 (112–185)
*General anesthesia*	2134 (96.5%)	1142 (64.3%)	209 (58.4%)
*Periprocedural complications*	2191 (99.1%)	–	–
*Dissections/perforations, n (%)*	–	44 (2.39%)	18 (4.85%)
*Intracranial hemorrhage on follow up image (any type), n (%)*	–	250 (13.6%)	47 (12.7%)
*Clinical outcome*	1951 (88.2%)	–	–
*mRS score at 90 days (median, Q1–Q3)*	–	3 (1–5)	5 (4–6)
*Mortality, n (%)*	–	397 (23.0%)	156 (44.2%)
*Good clinical outcome, n (%)*	–	663 (41.0%)	52 (15.5%)

*ICA* Internal Carotid Artery, *mRS* Modified Rankin Scale, *TICI* Thrombolysis in Cerebral Infarction

Of the 2211 patients who fulfilled the inclusion criteria, 1629 patients had complete datasets and were included in the multivariable analysis. Among these, 50.5% were female, and the mean age was 72.3 years (±13.0 years) (Table S1, supplemental material). The median NIHSS score on admission was 15 (range 10–18). The median ASPECTS on admission imaging was 9 (range 7–10). Concomitant stenting of cervical ICA was performed in 12.1% of patients. Failure of reperfusion occurred in 282/1629 cases (17.3%, Table S2, supplemental material).

### Procedural and Clinical Outcome

Failure to reach the thrombus (i.e. no thrombectomy maneuver performed) was reported in 30.2% of TICI 0‑1 patients. Of patients with successful reperfusion, 41% had a good clinical outcome. In contrast, only 15.5% of the patients with failed reperfusion achieved a good clinical outcome (mRS 0-2), with the proportion of good clinical outcome increasing with increasing TICI score (TICI 0: 8.4%, TICI 1: 17.6%, and TICI 2a: 25.2%).

### Multivariable Analysis

**Fig. 2:**
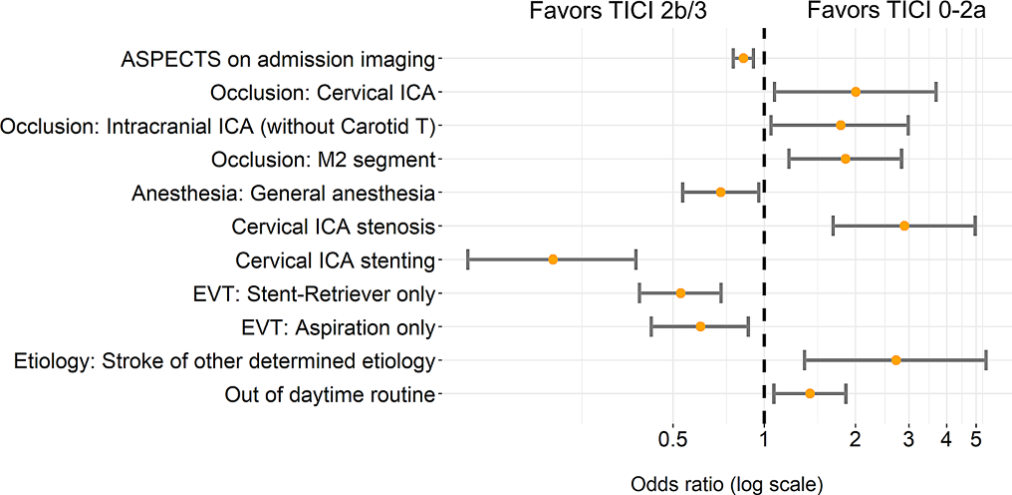
Adjusted odds ratio estimates with 95% confidence intervals for failure of reperfusion (Thrombolysis in Cerebral Infarction [TICI] 0-2a). Results are derived from a logistic regression model, adjusted for confounders (age, sex, admission National Institutes of Health Stroke Scale [NIHSS] score, prestroke modified Rankin Scale [mRS] score, admission imaging Alberta stroke program early CT score [ASPECTS], comorbidities, application of i.v. thrombolysis, location of vessel occlusion, stroke etiology, and treatment out of daytime routine), and based on 1629 complete observations. *ICA* Internal carotid artery, *M2* middle cerebral artery, *M2* segment, *EVT* endovascular therapy

Multivariable analysis was performed for the 1629 patients with complete datasets. Significant associations for failure of reperfusion (TICI 0-2a) are presented in Fig. 2, and the regression coefficients for all independent variables are included in Table 3.

**Table 3:** Results of logistic regression analysis with failure of reperfusion as dependent variable (*N* = 1629 complete datasets)

Variable	Odds ratio	95% CI low	95% CI high	*P* value
Age	1.01	1.00	1.02	0.126
Sex (female)	1.16	0.87	1.54	0.322
Baseline: prestroke mRS	1.13	1.00	1.27	0.051
Baseline: admission NIHSS	1.00	0.98	1.03	0.710
Comorbidity: arterial hypertension	0.90	0.65	1.27	0.553
Comorbidity: diabetes mellitus	1.07	0.75	1.51	0.715
Comorbidity: dyslipidemia	0.84	0.61	1.14	0.254
Comorbidity: atrial fibrillation	0.70	0.47	1.05	0.080
Left hemisphere	1.18	0.89	1.56	0.252
*ASPECTS on admission imaging*	*0.85*	*0.79*	*0.92*	*4.44* *×* *10*^*−5*^
Occluded vessel: intracranial ICA (carotid T)	1.29	0.85	1.95	0.228
*Occluded vessel: cervical (proximal) ICA*	*2.01*	*1.08*	*3.69*	*0.026*
*Occluded vessel: intracranial ICA (without carotid T)*	*1.79*	*1.05*	*2.98*	*0.029*
Occluded vessel: proximal middle cerebral artery, M1 segment	1.04	0.69	1.54	0.857
Occluded vessel: distal middle cerebral artery, M1 segment	0.93	0.60	1.42	0.740
*Occluded vessel: middle cerebral artery, M2*	*1.86*	*1.21*	*2.84*	*0.005*
intravenous thrombolysis	0.86	0.65	1.13	0.277
Anesthesia: emergency conversion to general anesthesia	0.90	0.42	1.80	0.767
*Anesthesia: general anesthesia*	*0.72*	*0.54*	*0.96*	*0.025*
*Cervical ICA stenosis (>70%)*	*2.90*	*1.69*	*4.97*	*1.05* *×* *10*^*−4*^
*Cervical ICA stenting*	*0.20*	*0.11*	*0.38*	*7.66* *×* *10*^*−7*^
*EVT: stent-retriever only*	*0.53*	*0.39*	*0.72*	*5.93* *×* *10*^*−5*^
*EVT: aspiration only*	*0.62*	*0.42*	*0.89*	*0.010*
Etiology: cardioembolic stroke	1.12	0.68	1.84	0.656
Etiology: dissection	1.10	0.38	2.91	0.850
Etiology: large artery atherosclerosis	1.53	0.97	2.44	0.070
*Etiology: other determined etiology*	*2.73*	*1.36*	*5.39*	*0.004*
*Treatment outside of daytime routine*	*1.41*	*1.07*	*1.86*	*0.013*

*ASPECTS* Alberta stroke program early CT score, *EVT* Endovascular therapy, *ICA* internal carotid artery, *mRS* modified Rankin Scale, *NIHSS* National Institutes of Health Stroke Scale

A positive association with reperfusion failure was found for complete occlusion of cervical ICA (adjusted OR 2.01, 95% CI 1.08–3.69), occlusion of intracranial ICA without carotid T (adjusted OR 1.79, 95% CI 1.05–2.98), occlusion of M2 segment (adjusted OR 1.86, 95% CI 1.21–2.84), proximal ICA stenosis (adjusted OR 2.90, 95% CI 1.69–4.97), stroke of other determined etiology according to the TOAST classification [9] (e.g. vasculopathies or hypercoagulable states, adjusted OR 2.73, 95% CI 1.36–5.39), and treatment outside of routine clinical hours (adjusted OR 1.41, 95% CI 1.07–1.86). A negative association with reperfusion failure was found for initial higher ASPECTS (adjusted OR per ASPECTS point 0.85, 95% CI 0.79–0.92), treatment with the patient under general anesthesia (adjusted OR 0.72, 95% CI 0.54–0.96), concomitant stenting of ICA stenosis (adjusted OR 0.20, 95% CI 0.11–0.38), and EVT exclusively with stent-retriever devices (adjusted OR 0.53, 95% CI 0.39–0.72), or exclusively with aspiration devices (adjusted OR 0.62, 95% CI 0.42–0.89).

An extension of the multivariable model that additionally included the time from symptom onset to groin puncture was created using data from 966 patients. Time from symptom onset to groin puncture was not associated with failure of reperfusion (adjusted OR 1.00, 95% CI 1.00–1.00).

### Early Termination vs. Multiple Reperfusion Attempts

In 58 patients treatment was stopped after 1 single EVT attempt, despite reperfusion failure. For this collective, the mean age was 73.1 years (SD 12.6 years) and M2 occlusions were the most frequent location of occlusion (*n* = 22/58, 37.9%), followed by proximal M1 occlusions (*n* = 20, 34.5%). Intravenous thrombolysis was administered in 35/58 patients (61.4%). The median NIHSS score at admission was 14 (8–19). The median mRS score at day 90 was 4.5 (2–6). A good clinical outcome was observed in 16 patients (32.0%).

In 56 patients with a mean age of 72.1 years (SD 13.8), reperfusion failed despite 5 or more EVT attempts. Carotid T occlusions were the most frequent in this group (*n* = 21, 37.5%), followed by proximal M1 occlusions (*n* = 18/56, 32.1%). Intravenous thrombolysis was administered in 21/56 patients (38.9%). The median NIHSS score on admission was 16 (14–20). The median mRS score at day 90 was 6 (4–6). A good clinical outcome was observed in only 3 patients (5.7%).

## Discussion

The present retrospective study aimed at identifying factors associated with failure of EVT to achieve TICI 2b/3 reperfusion in a large multicenter dataset of acute stroke patients. In this cohort, 16.8% of patients had failed EVT (TICI 0/1/2a) following intervention, which is at the lower end of the spectrum when compared to other randomized controlled trials (failure of reperfusion varying between 12% and 41% of all patients) [10–14]. Large cohort registries reported between 12% and 20% of patients having TICI 0-2a [15, 16].

Other retrospective studies reported failure to reach the thrombus (i.e. failure to reach the occlusion or failure to pass the thrombus with the microcatheter) in roughly 45% of patients with TICI 0‑1 [3, 4]. In order to ensure comparability with these studies, this study analyzed the particular item failure to reach the thrombus in patients with TICI 0‑1 (not including TICI 2a). Failure to reach the thrombus was found in 30.2% of patients with TICI 0‑1, which is slightly lower but comparable to the rates found in the literature [3, 4]. Hence, even when EVT proves unsuccessful, the majority of intracranial occlusions can be accessed.

In the present study, the occlusion site was associated with failure of reperfusion in the multivariable analysis and M2 occlusions were more likely to be associated with reperfusion failure, as has been previously described [4]; however, the results do not imply that achieving reperfusion in the M2 segment is necessarily more difficult than in the M1 segment. A possible explanation is that M2 occlusions cause less severe clinical symptoms, especially in the case of good collaterals, making it more likely for the interventionalist to prematurely terminate EVT for an M2 occlusion, despite failure of reperfusion. This is illustrated by the analysis of early aborted procedures (defined by a single retrieval attempt despite failed reperfusion), where M2 occlusions were the most frequent type. Furthermore, cervical ICA occlusions and intracranial ICA occlusions (without the carotid T) exhibited the lowest rate of sufficient reperfusion and were both associated with failure of reperfusion. A possible explanation is that occlusions of the cervical ICA and intracranial ICA (without the carotid T) are more frequently caused by or combined with severe arteriosclerotic changes or dissections, which in turn complicate EVT.

Unfortunately, the data do not enable a differentiation between singular ICA occlusions and those with tandem lesions; however, a comparably low sufficient reperfusion rate of 70% for cervical ICA occlusions has been previously described, both in a multicenter retrospective study of 305 patients with tandem occlusions [17] and in a retrospective study of 121 proximal ICA (tandem) occlusions [18]. Conversely, another retrospective study with 163 patients with tandem occlusion reported a 91% rate of sufficient reperfusion [19]. These differences could be explained by the chosen definition of tandem occlusion (high-grade stenosis vs. complete occlusion), as well as the rate of concomitant ICA stenting.

Significant stenosis of the cervical ICA was an independent predictor of reperfusion failure. A possible explanation could be high-grade stenosis inhibiting intracranial access resistant to proximal stenting or associated intracranial arteriosclerotic disease. On the other hand, increased odds of successful reperfusion were found whenever a stent was placed in the proximal ICA, which has also been previously described [20–22]: however, in this group a selection bias is likely: stent placement can only be performed if access is possible and is much more likely to be performed in patients with successful intracranial reperfusion.

In line with another study by Tsang et al. no differences were found regarding failure of reperfusion between stroke due to large-vessel atherosclerosis and those of cardioembolic origin [23]. In this study, however, stroke of other determined etiology, as defined by the TOAST criteria [9], were associated to a greater extent with reperfusion failure. This suggests that rare etiologies of stroke, e.g. nonatherosclerotic vasculopathies and hypercoagulable states, might be more difficult to treat with EVT or that treatment is often terminated at an earlier timepoint in these patients. Interestingly, a retrospective study on i.v. thrombolysis found better outcomes in patients with large-vessel atherosclerosis and cardioembolic strokes, compared to strokes of other determined etiology [24].

An association was found between lower baseline admission ASPECTS and failure of reperfusion. It could be hypothesized that EVT is terminated early in patients with low ASPECTS; however, no difference was found between the admission ASPECTS of patients with early abortion vs. those with multiple retrieval attempts. The poor reperfusion results in patients with low ASPECTS are more likely caused by poor collaterals, which are independently associated with reperfusion failure [25].

In the present study, the exclusive use of stent-retriever or aspiration devices were both associated with sufficient reperfusion. This is most likely explained by the fact that difficult cases with insufficient reperfusion often involve the employment of multiple techniques. Furthermore, no differences were found in reperfusion success between patients treated exclusively with stent-retrievers or those treated with aspiration devices, as has also been reported by the COMPASS trial [26], as well as by a recent meta-analysis [23]. In this context, it is of importance to note that recent studies have demonstrated that a combined approach, such as stent retriever-assisted vacuum-locked extraction (SAVE) appears to be superior in achieving reperfusion with a single pass [36].

In this study, general anesthesia was associated with a higher reperfusion rate when compared to conscious sedation. This finding has been previously described in a RCT [27]; however, a large systematic review and meta-analysis of 6703 patients did not find a difference between anesthesia type and rates of sufficient reperfusion [28]. This interesting aspect should be further explored in future trials, since the decision for or against general anesthesia is frequently encountered in daily practice [29].

In this study, patients treated outside of routine clinical hours had a slight yet significant risk increase for failure of reperfusion. The reasons for this are beyond the scope of this study; further research is required to confirm this finding, as well as to investigate its possible causes. An explanation could be the presence of less experienced operators during nighttime shifts. Furthermore, delayed transfer times of EVT patients has been associated with nighttime operating hours [30]. This study, however, did not find an association between time from symptom onset to final angiographic run and reperfusion result. A weekend effect has been described in the pre-EVT era and has been defined as a worse clinical outcome for patients treated for acute ischemic stroke outside of routine daytime hours [31] and has been reported for patients with EVT as well [32]; however, other studies focusing on patients treated with EVT did not describe such an effect [33–35].

Early treatment termination was found for a large proportion of M2 occlusions with i.v. thrombolysis, suggesting that interventionalists deliberately decided to stop treatment earlier in patients with peripheral occlusions, who potentially benefit from i.v. thrombolysis. In contrast, multiple reperfusion attempts were performed for more proximal occlusions (carotid T and M1) in patients with low rates of i.v. thrombolysis (most likely due to contraindications). In such cases, EVT is the preferred treatment. Patients with carotid T occlusions are of clinical importance since they tend to have very poor outcomes when recanalization is not achieved.

The results of the present study need to be interpreted with caution. The image analysis and classification of final TICI score was self-adjudicated by each center, presenting a major limitation. Furthermore, the patient group exhibiting reperfusion failure is heterogeneous. In some patients, treatment was stopped early (without any EVT attempt or after a single attempt), while in others, treatment failed despite multiple attempts. These two collectives cannot be directly compared, and many patients are likely somewhere in between. The retrospective nature of the analysis also makes it difficult to differentiate between technical difficulties and deliberate termination of EVT. Furthermore, patients were included between July 2015 and April 2018, and experience, technical approaches, and thrombectomy selection criteria may have changed during this time. Only 1629 of 2211 patients had complete datasets but the baseline data as well as rate of reperfusion, were comparable to those of the entire patient cohort. Some variables were only available in an even smaller subset of patients, especially time from symptom onset to admission and time from groin puncture to final TICI score. Further limitations include the possible heterogeneity of data due to multicenter data acquisition, limited details on thrombectomy technique and devices used, and the limited number of patients included in the multiple regression analysis due to missing values.

## Conclusion

Several factors associated with failure of reperfusion were identified, most notably occlusions of the cervical and proximal ICA and lower baseline ASPECTS. Conversely, stent placement in the proximal ICA was associated with reperfusion success.

## Caption Electronic Supplementary Material

Table S1 and S2 show the baseline and outcome data for patients with complete datasets that were included in the multivariable analysis (*n* = 1629)
