# Supplementary material for: Factors Associated with Failure of Reperfusion in Endovascular Therapy for Acute Ischemic Stroke: A Multicenter Analysis
Source: Clin Neuroradiol. 2020 Feb 17;31(1):197–205. doi: 10.1007/s00062-020-00880-8 (PMC7943507; doi:10.1007/s00062-020-00880-8)
Supplement: Supplementary file 1 — Table S1 and S2 show the baseline and outcome data for patients with complete datasets that were included in the multivariable analysis (n = 1629) [file 62_2020_880_MOESM1_ESM.docx]

**Table S1: Baseline clinical data for patients included in multivariable analysis (n=1629)**

| **Variable** | **Sufficient reperfusion (TICI 2b-3, n=1347)** | **Insufficient reperfusion (TICI 0/1/2a, n=282)** |
| --- | --- | --- |
| **Age, years (mean, SD)** | 72.2 (13.0) | 72.8 (12.7) |
| **Female sex, n (%)** | 675 (50.1%) | 148 (52.5%) |
| **Hypertension, n (%)** | 1011 (75.1%) | 207 (73.4%) |
| **Diabetes mellitus, n (%)** | 259 (19.2%) | 56 (19.9%) |
| **Dyslipidemia, n (%)** | 438 (32.5%) | 75 (26.6%) |
| **Atrial fibrillation, n (%)** | 565 (41.9%) | 94 (33.3%) |
| **Initial NIHSS score (median, Q1-Q3)** | 15 [10-18] | 15 [10-19] |
| **Initial ASPECTS (median, Q1-Q3)*** | 9 [7-10] | 8 [6-9] |
| **Initial occlusion site, n (%)** |  |  |
| **Left hemisphere*** | 697 (51.7%) | 155 (54.9%) |
| **Location of vessel occlusion** |  |  |
| ICA (cervical) | 72 (5.35%) | 25 (8.87%) |
| ICA (non Carotid-T) | 77 (5.72%) | 29 (10.3%) |
| Carotid-T | 243 (18.0%) | 65 (23.0%) |
| M1 proximal | 525 (39.0%) | 100 (35.5%) |
| M1 distal | 326 (24.2%) | 56 (19.9%) |
| M2 | 309 (22.9%) | 78 (27.7%) |
| **Intravenous tPA, n (%)** | 775 (57.5%) | 151 (53.5%) |
| **Onset to admission, min (median, Q1-Q3)** | 128 [60.0-200] | 140 [59.5-208] |
| **Treatment out of daytime routine (Monday to Friday, 8 a.m. to 5 p.m.)** | 515 (38.2%) | 131 (46.4%) |
| **Stroke etiology** |  |  |
| Cardioembolism | 715 (53.1%) | 121 (42.9%) |
| Dissection | 26 (1.93%) | 7 (2.48%) |
| Atherosclerosis | 342 (25.4%) | 97 (34.4%) |
| Other determined etiology | 43 (3.19%) | 19 (6.47%) |
| Unknown etiology | 221 (16.4%) | 38 (13.5%) |

**Table S2: Procedural and clinical outcome for patients included in multivariable analysis (n=1629)**

| **Variable** | **Sufficient reperfusion (TICI 2b-3, n=1840)** | **No, minimal reperfusion or partly reperfusion (TICI 0/1/2a, n=371)** |
| --- | --- | --- |
| **Final TICI score** |  |  |
| **0** | 0 (0.00%) | 142 (50.4%) |
| **1** | 0 (0.00%) | 34 (12.1%) |
| **2a** | 0 (0.00%) | 106 (37.6%) |
| **2b** | 606 (45.0%) | 0 (0.00%) |
| **3** | 741 (55.0%) | 0 (0.00%) |
| **Proximal ICA stenosis >70% on angiogram, n (%)** | 208 (15.4%) | 64 (22.7%) |
| **Concomitant ICA stenting** | 168 (12.5%) | 29 (10.3%) |
| **Time from admission to groin puncture (median, Q1-Q3)** | 72 [51-100] | 77 [53-112] |
| **Time from groin puncture to final TICI (median, Q1-Q3)** | 119 [90-156] | 134 [113-187] |
| **General anesthesia** | 871 (64.7%) | 161 (57.1%) |
| **Periprocedural complications** |  |  |
| **Dissections/Perforations, n (%)** | 34 (2.5%) | 17 (6.0%) |
| **Intracranial hemorrhage on follow up image (any type), n (%)** | 192 (14.3%) | 35 (12.4%) |
| **Clinical outcome** |  |  |
| **mRS at 90 days (median, Q1-Q3)** | 3 [1-5] | 5 [3-6] |
| **Mortality, n (%)** | 267/1170  (21.0%) | 111/257  (42.2%) |
| **Good clinical outcome, n (%)** | 495/1170  (42.3%) | 43/257  (16.7%) |
